# Availability of orchid mycorrhizal fungi on roadside trees in a tropical urban landscape

**DOI:** 10.1038/s41598-019-56049-y

**Published:** 2019-12-20

**Authors:** Muhammad Izuddin, Amrita Srivathsan, Ai Lan Lee, Tim Wing Yam, Edward L. Webb

**Affiliations:** 1 Department of Biological Sciences, National University of Singapore, 14 Science Drive 4, Singapore, 117543 Singapore; 2 Singapore Botanic Gardens, 1 Cluny Road, Singapore, 25956 Singapore

**Keywords:** Conservation biology, Urban ecology

## Abstract

Urban expansion threatens biodiversity worldwide, therefore urban spaces need to be amenable to biodiversity conservation. On trees in urban environments, natural colonisation and successful translocation of epiphytic orchids are necessary to enhance urban biodiversity, and depend on the availability of compatible orchid mycorrhizal fungi (OMF). However, the extent of OMF presence and distribution, as well as niche requirements for the OMF, remain poorly studied. To identify and quantify OMF on urban trees as well as assess their suitability for native epiphytic orchids, we conducted high-throughput sequencing on tree bark and orchid root samples. OMF were detected at 60% of the study sites on 16% of 270 bark samples (from stem, fork, and branch microsites within each tree). OMF presence and richness on bark samples were related to multiple biophysical factors; in general, humus presence and precipitation levels were positively predictive of OMF presence and richness. We found Ceratobasidiaceae- and Serendipitaceae-associated OMF both on bark and within roots. Orchid species also showed differing mycorrhizal specificity. Sites associated with fungal genera *Ceratobasidium*, *Rhizoctonia*, and *Serendipita* were considered suitable habitats for seven orchid species. The results suggest that urban trees support OMF and are therefore suitable for native orchid species; however, OMF availability are largely constrained by biophysical factors. To maximise the likelihood of translocation success and consequent natural establishment, we propose that (micro)sites are screened for compatible OMF prior to any intervention.

## Introduction

Urbanisation is continually imposing extensive pressures on biodiversity worldwide. With the increase in global population size and an increased tendency towards residence in urban areas, the risk of biodiversity loss continues to rise^1,2^. Urban expansion is projected to occur near protected areas and biodiversity hotspots, notably in developing regions such as Southeast Asia, China, and South America^3^. Urbanisation may lead to habitat degradation, fragmentation, and/or loss, which consequently lead to demographic or genetic isolation as well as species extirpation^4,5^. Indeed, urbanisation has induced substantial biodiversity loss; therefore careful management and conservation strategies in urban spaces are required to contribute to biodiversity conservation^6–9^. With urban areas becoming increasingly widespread, the need to assess the capacity of these spaces for biodiversity conservation becomes more urgent, necessitating knowledge of taxa and sites that are amenable to conservation in non-forest habitats.

Because of habitat loss and over-collection, one of the richest plant families worldwide, Orchidaceae, is also one of the most endangered^10–12^. Orchids are sensitive to anthropogenic environmental changes, chiefly because of their strong reliance on other taxa such as unique pollinators and mycorrhizal fungi^13–16^. Despite this, orchids have been found to colonise urban trees, indicating potential suitability for conservation in non-natural landscapes^17–19^. Furthermore, some orchids are amenable to *ex situ* conservation in non-forest habitats^20–23^. To improve the likelihood of successful *ex situ* efforts, detailed assessments regarding habitat limitations and requirements are necessary.

Orchid mycorrhizal fungi (OMF) play a pivotal role in determining orchid establishment, survival, and development^13,24^, especially for epiphytic species that often establish under water and nutrient-scarce conditions^25,26^. By forming obligate relationships with compatible fungi, orchid hosts gain carbon, nutrients, and water at one or more life history stages^13,27^. The majority of orchids associate with fungal taxa belonging to the polyphyletic/“rhizoctonia” group of Basidiomycetes, specifically Ceratobasidiaceae, Serendipitaceae (Sebacinales), and Tulasnellaceae^13,28,29^. Identifying specific OMF may be advantageous for orchid conservation—e.g., orchid propagation, *ex situ* seeding, seedling translocation—as contrasting fungal taxa may differ in nutrient uptake efficiency or stimulation of germination^30–33^.

Even though orchids show strong dependence on mycorrhizal fungi, OMF presence and distribution are independent of orchid distribution^34,35^. Indeed most research has inferred OMF presence and spatial distribution from orchid roots^35–38^. While root-based inferences can help determine species-specific mycorrhizal associations, they do not directly inform the extent of OMF presence and distribution^13^. With the advent of high-throughput sequencing technologies, microbial communities, diversity, and biogeography can be assessed at high resolutions^39,40^. High-throughput sequencing allows recovery of microbial amplicons, including unculturable microorganisms, from environmental samples such as soil and tree bark^41^. In this context, high-throughput sequencing can facilitate large-scale screening for known OMF on potential (micro)sites. Variation in biophysical conditions are also reported to influence OMF presence and distribution (i.e. niche requirements)^31,42^. Moreover, effects of specific biophysical factors (e.g., ambient temperature, relative humidity) may vary between micro/local and macro/landscape scale^43,44^. Fundamentally, the availability of suitable OMF and niche conditions are imperative for both *in situ* as well as *ex situ* conservation to succeed^24,45–48^.

Singapore has undergone massive land transformation (>99% of its original forest cover transformed), making it one of the most modified countries in the world^49,50^. To address vegetation and species loss, the city-state adopts multifarious policies and programmes that are dedicated to urban greening^51–53^. The progressive nature of the country’s urban-green matrix makes Singapore an apt study model for developing nations and cities in relation to biodiversity conservation in the urban landscape (e.g., habitat enrichment, managed relocation). The “orchid conservation programme” was one of many schemes administered by the Singapore government to address vegetation and species loss; this programme entailed experimental as well as restoration plantings of native epiphytic orchids—including nationally Endangered and Presumed Nationally Extinct species—on multiple sites, mainly urban parks^23,52^. Between 2009 and 2011, seedlings were propagated asymbiotically and planted on microsites (i.e. stem, fork, and branch) of various host trees, mostly *Albizia saman* (Jacq.) Merr. trees^21^. Following translocation, various orchid species formed symbioses with compatible OMF that were available on the host trees^20,24^. These translocated orchids provide an opportunity for the detection and identification of potential orchid-fungal associations. This is especially valuable for native species that are naturally rare or extinct. Unique circumstance such as this can provide both qualitative and empirical data on relevant OMF, and thereby, help devise decision-making frameworks that can facilitate the identification of alternative (micro)sites that may be applicable to future conservation attempts^54,55^.

In this study, we investigated whether OMF are available on urban roadside trees. To get an overview of the presence and distribution of OMF on urban sites as well as their suitability for native epiphytic orchids (i.e. presence of compatible OMF), we assessed the mycorrhizal communities of both the tree bark (microsites) and roots of translocated orchid species (from the abovementioned “orchid conservation programme”) by using DNA barcodes and high-throughput (Illumina® MiSeq) sequencing technology. We also investigated possible biophysical factors that could influence OMF presence and richness on urban sites. As previous studies have reported orchid colonisations in urban environments, we expected OMF to be present on urban trees^18,19^. We also expected variations in OMF presence to be related to specific biophysical factors. Knowledge of OMF availability and associated niche requirements can help with the selection of suitable (micro)sites for future orchid conservation efforts.

## Results

**Figure 1:**
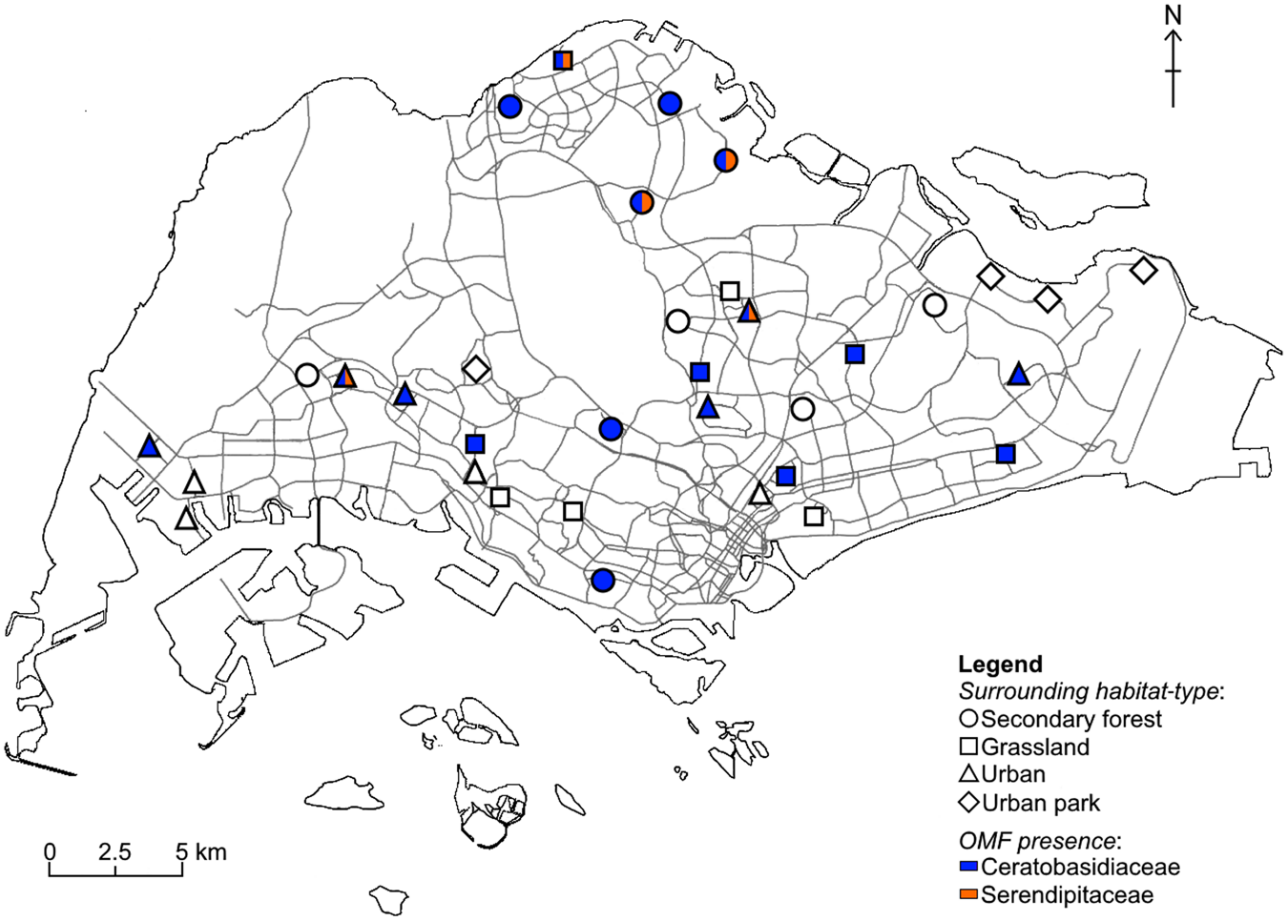
Map of all bark and root sampling locations as well as geographical distribution of orchid mycorrhizal fungi (OMF) on roadside trees.

Overall, ~41.7 million reads were obtained and assigned to 270 bark samples and 65 root samples (average number of reads per sample = 80,396 for bark samples and 308,289 for root samples). After quality-filtering, each bark and root sample contained 0–36 and 0–48 unique sequences respectively. Clustering sequences to OTUs yielded 593 OTUs (6,270 unique sequences), of which 26 were assigned to putative OMF-OTUs in three families: Ceratobasidiaceae (8 OMF-OTUs), Serendipitaceae (10 OMF-OTUs), and Tulasnellaceae (8 OMF-OTUs). OMF-OTUs were found on 43 of 270 (16%) of bark samples/microsites (Table 1), or more broadly, 18 out of 30 sites (Fig. 1). We also successfully retrieved OMF-OTUs from roots of 8 of the 11 orchid species sampled (Table 2; see also Supplementary Table S1 The Ceratobasidiaceae- and Serendipitaceae-related sequences were present across both bark and root samples, whereas Tulasnellaceae-related sequences were detected only in orchid roots. The ranges of OMF-OTUs were 0–3 per microsite (mean = 0.2), 0–6 per site (mean = 1.6), and 0–10 per orchid species (mean = 2.0). Non-rhizoctonia OTUs encountered on bark samples and in orchid roots were largely Ascomycetes, e.g., genera *Fusarium* (Hypocreales), *Lachnum* (Helotiales), and *Curvularia* (Pleosporales); basidiomycete representatives such as genera *Mycena* (Agaricales) and *Marasmius* (Agaricales) were also detected.

**Table 1:** List of fungal operational taxonomic units (OTUs)^a^ identified on bark samples using sequencing. Orchid-site suitability was assessed at genus-level (see Table 2 and Fig. 1).

OTU	Length (bp) [n = frequency of occurrence]	Phylogenetic relationship^b^			
		Taxonomic affiliation	Closest match in GenBank (accession number)	Sequence identity (%)	Suitable orchid species
OTU100	243 [n = 24]	Ceratobasidiaceae	*Ceratobasidium cereale* (KT362077)	97	*Cb*^†^; *Cr*^‡^
OTU102	357 [n = 3]	Ceratobasidiaceae	*Ceratobasidium cereale* (MF471701)	100	*Cb*^†^; *Cr*^‡^
OTU148	325 [n = 11]	Ceratobasidiaceae	*Ceratobasidium* sp. AG-P (KP125334)	100	*Cb*^†^; *Cr*^‡^
OTU159	336 [n = 1]	Ceratobasidiaceae	*Rhizoctonia solani* (KM488565)	96	*Cr*^‡^; *Pc*^‡^
OTU183	302 [n = 3]	Ceratobasidiaceae	*Ceratobasidium theobromae* (KU319573)	88	*Cb*^†^; *Cr*^‡^
OTU262	329 [n = 2]	Ceratobasidiaceae	*Ceratobasidium* sp. AG-R (KY880973)	99	*Cb*^†^; *Cr*^‡^
OTU366	293 [n = 1]	Ceratobasidiaceae	*Ceratobasidium cereale* (MF471701)	91	*Cb*^†^; *Cr*^‡^
OTU86	302 [n = 12]	Serendipitaceae	Sebacinaceae sp. 11 MB-2012 (JX138554)	98	*—*
OTU195	391 [n = 3]	Serendipitaceae	*Serendipita vermifera* (FN663149)	98	*Bm*^‡^, *Cb*^†^, *Cf*^†^, *Da*^‡^, *Dl*^‡^
OTU270	269 [n = 1]	Serendipitaceae	*Serendipita vermifera* (FN663149)	97	*Bm*^‡^, *Cb*^†^, *Cf*^†^, *Da*^‡^, *Dl*^‡^
OTU392	313 [n = 1]	Serendipitaceae	*Serendipita* sp. MAFF 305839 (KF061290)	95	*Bm*^‡^, *Cb*^†^, *Cf*^†^, *Da*^‡^, *Dl*^‡^
OTU396	317 [n = 1]	Serendipitaceae	*Serendipita* sp. MAFF 305839 (KF061290)	95	*Bm*^‡^, *Cb*^†^, *Cf*^†^, *Da*^‡^, *Dl*^‡^

^a^OTUs were defined by 97% internal transcribed spacer (ITS) sequence similarity; ^b^Based on BLAST analysis (October 2017); *Bm*: *Bulbophyllum medusae* (Lindl.) Rchb. f., *Cb*: *Cymbidium bicolor* ssp. Lindl. ssp. *pubescens* (Lindl.) Du Pay & Cribb, *Cf*: *Cymbidium finlaysonianum* Lindl., *Cr*: *Coelogyne rochussenii* de Vr., *Da*: *Dendrobium aloifoliuim* (Blume) Rchb. f., *Dl*: *Dendrobium leonis* (Lindl.) Rchb. f., *Pc*: *Phalaenopsis cornu-cervi* (Breda) Blume & Rchb. f.; statuses of orchid species: ^†^nationally Critically Endangered or ^‡^Presumed Nationally Extinct.

**Table 2:** List of fungal operational taxonomic units (OTUs)^a^ identified in orchid roots using sequencing.

OTU	Length (bp) [n = frequency of occurrence]	Phylogenetic relationship^b^			
		Taxonomic affiliation	Closest match in GenBank (accession number)	Sequence identity (%)	Associated orchid species
OTU100	243 [n = 1]	Ceratobasidiaceae	*Ceratobasidium cereale* (KT362077)	97	*Cb*^†^
OTU159	336 [n = 1]	Ceratobasidiaceae	*Rhizoctonia solani* (KM488565)	96	*Cr*^‡^
OTU183	302 [n = 1]	Ceratobasidiaceae	*Ceratobasidium theobromae* (KU319573)	88	*Cr*^‡^
OTU438	251 [n = 1]	Ceratobasidiaceae	*Rhizoctonia solani* (JQ676901)	100	*Pc*^‡^
OTU548	291 [n = 3]	Serendipitaceae	Sebacinales sp. 44 (HQ853682)	95	*Cr*^‡^
OTU549	303 [n = 1]	Serendipitaceae	Sebacinales sp. 4035 (JF906112)	89	*Dl*^‡^
OTU550	314 [n = 5]	Serendipitaceae	*Sebacina* [ = *Serendipita*] sp. Seb25I (AB831811)	90	*Bm*^‡^; *Cb*^†^; *Cf*^†^, *Da*^‡^
OTU560	303 [n = 1]	Serendipitaceae	*Sebacina* [ = *Serendipita*] sp. (KX185541)	91	*Dl*^‡^
OTU561	305 [n = 2]	Serendipitaceae	*Sebacina* [ = *Serendipita*] sp. (KC928372)	90	*Dl*^‡^
OTU574	527 [n = 1]	Tulasnellaceae	*Epulorhiza* sp. M-1 (JQ713573)	85	*Dl*^‡^
OTU575	527 [n = 4]	Tulasnellaceae	*Tulasnella* sp. DC225 (KC152330)	90	*Dl*^‡^
OTU576	527 [n = 3]	Tulasnellaceae	*Tulasnella* sp. DC225 (KC152326)	91	*Dl*^‡^
OTU579	527 [n = 2]	Tulasnellaceae	*Tulasnella* sp. DC225 (KC152330)	92	*Cm*^‡^; *Dl*^‡^
OTU582	527 [n = 1]	Tulasnellaceae	*Tulasnella* sp. DC225 (KC152330)	87	*Dl*^‡^
OTU585	540 [n = 1]	Tulasnellaceae	*Tulasnella* sp. DC225 (KC152324)	90	*Dl*^‡^
OTU589	471 [n = 1]	Tulasnellaceae	*Tulasnella* sp. DC225 (KC152333)	91	*Dl*^‡^
OTU591	527 [n = 1]	Tulasnellaceae	Tulasnellaceae sp. 7 MB-2012 (JX138563)	97	*Dl*^‡^

^a^OTUs were defined by 97% internal transcribed spacer (ITS) sequence similarity; ^b^Based on BLAST analysis (October 2017); *Bm*: *Bulbophyllum medusae* (Lindl.) Rchb. f., *Cb*: *Cymbidium bicolor* ssp. Lindl. ssp. *pubescens* (Lindl.) Du Pay & Cribb, *Cf*: *Cymbidium finlaysonianum* Lindl., *Cm*: *Coelogyne mayeriana* Rchb. f., *Cr*: *Coelogyne rochussenii* de Vr., *Da*: *Dendrobium aloifoliuim* (Blume) Rchb. f., *Dl*: *Dendrobium leonis* (Lindl.) Rchb. f., *Pc*: *Phalaenopsis cornu-cervi* (Breda) Blume & Rchb. f.; statuses of orchid species: ^†^nationally Critically Endangered or ^‡^Presumed Nationally Extinct.

**Figure 2:**
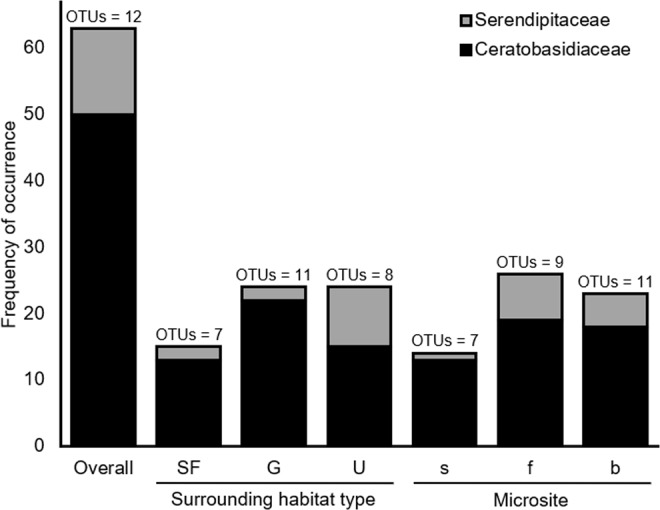
Frequency of occurrence and richness of orchid mycorrhizal fungus OTUs (operational taxonomic units) on urban roadside trees in Singapore, disaggregated by surrounding habitat-type and microsite-type: SF, secondary forest, G, grassland, U, urban, s, stem, f, fork, and b, branch.

**Figure 3:**
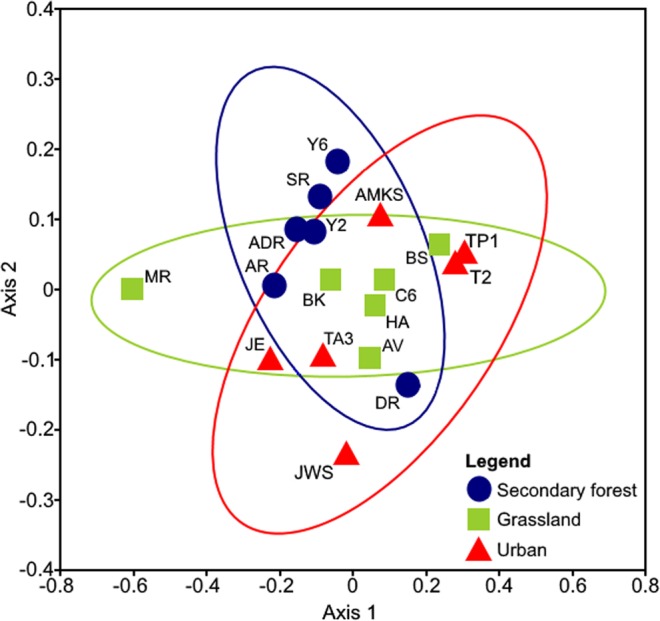
Non-metric multidimensional scaling (NMDS) plot of orchid mycorrhizal fungi detected on bark samples from three different habitat-types (stress value = 0.245). The 95% confidence interval ellipses are shown.

The composition of OMF varied between sites. The more common Ceratobasidiaceae was present at 18 sites, of which five had Serendipitaceae present as well (Fig. 1). Both “grassland” and “urban” sites had the highest frequency of OMF occurrence while the former had the highest OMF richness (Fig. 2). “Secondary forest” sites had the lowest frequency of OMF occurrence and richness. The NMDS showed differentiation in fungal community composition between the three habitat-types, particularly “secondary forest” and “grassland” sites (Fig. 3), which was statistically supported by ANOSIM test (Bray-Curtis; *R* = 0.16, *P* = 0.04).

The overall OMF richness across microsites was 12 OMF-OTUs (Richness_Chao2_ = 17.0 ± 8.1); the closeness of the OMF richness estimate (Chao2 diversity estimator) suggested that our sampling effort (n = 126) was adequate. The fork microsites had the highest frequency of OMF occurrence whereas the branch microsites had the highest OMF richness (Fig. 2). The stem microsites had the lowest frequency of OMF occurrence and richness.

Members of Ceratobasidiaceae (4 OMF-OTUs), Serendipitaceae (5 OMF-OTUs), and Tulasnellaceae (8 OMF-OTUs) occurred in the roots of eight orchid species (Table 2). Three species were associated with representatives of Ceratobasidiaceae, six with Serendipitaceae, and two with Tulasnellaceae. In addition, two orchid species were associated with more than one rhizoctonia family: *Dendrobium leonis* (Lindl.) Rchb. f. with Serendipitaceae and Tulasnellaceae, and *Cymbidium bicolor* Lindl. ssp. *pubescens* (Lindl.) Du Pay & Cribb with Ceratobasidiaceae and Serendipitaceae. Five orchid species were associated with one OMF-OTU except *D. leonis* (11 OMF-OTUs), *Coelogyne rochussenii* de Vr. (3 OMF-OTUs), and *C. bicolor* (2 OMF-OTUs) (Table 2). Based on genus similarity between bark- and root-associated OMF-OTUs (i.e. orchid-site suitability assessment), sites associated with members of *Ceratobasidium* (Ceratobasidiaceae) were considered as potential habitats for the orchid species *C. bicolor* and *C. rochussenii*, *Rhizoctonia-*associated (Ceratobasidiaceae) sites for *C. rochussenii* and *Phalaenopsis cornu-cervi* (Breda) Blume & Rchb. f., and *Serendipita-*associated (Serendipitaceae) sites for *Bulbophyllum medusae* (Lindl.) Rchb. f., *C. bicolor*, *Cymbidium finlaysonianum* Lindl., *Dendrobium aloifolium* (Blume) Rchb. f., and *D. leonis* (see Table 1).

At the microsite scale, habitat-type corresponded to OMF presence (Table 3). Mean daily rainfall and presence of humus were positively predictive of both OMF presence and richness. Similarly, humus presence positively influenced presence and richness of Ceratobasidiaceae-related OMF. For Serendipitaceae, OMF presence showed a positive association with humus presence while OMF richness positively corresponded to presence of moss and mean daily rainfall. At the site scale, the overall model indicated the positive relationship between frequency of OMF occurrence and mean daily rainfall. The same trend held true when considering only Serendipitaceae-related OMF. By contrast, no biophysical factor was predictive of Ceratobasidiaceae-related OMF occurrence. No spatial autocorrelation was detected in all data sets.

**Table 3:** Results of generalised linear models (GLMs) and generalised linear mixed models (GLMMs) illustrating the biophysical variables that were influential predictors of orchid mycorrhizal fungus (OMF) presence and richness at microsite-level and frequency of OMF occurrence at site-level.

Biophysical variables	Estimate	SE	*P*-value
Microsite-level			
Overall			
Presence^†b^			
Habitat-type/surrounding land use	—	—	<0.01
Presence of humus	1.275	0.380	<0.001
Mean daily rainfall	0.934	0.337	<0.01
Richness^‡p^			
Presence of humus	0.974	0.331	<0.01
Mean daily rainfall	0.826	0.365	<0.05
Ceratobasidiaceae			
Presence^†b^			
Presence of humus	0.858	0.374	<0.05
Richness^‡p^			
Presence of humus	0.755	0.355	<0.05
Serendipitaceae			
Presence^‡b^			
Presence of humus	8.773	4.167	<0.05
Richness^‡p^			
Presence of moss	1.933	0.806	<0.05
Mean daily rainfall	4.766	2.098	<0.05
Site-level			
Overall			
Frequency of occurrence^†p^			
Mean daily rainfall	0.885	0.342	<0.05
Serendipitaceae			
Frequency of occurrence^†p^			
Mean daily rainfall	3.952	1.232	<0.01

No predictor was influential in determining the frequency of occurrence of Ceratobasidiaceae at site-level. OMF presence models were fitted with a binomial or quasi-binomial error structure whereas OMF richness and frequency of occurrence models were fitted with a Poisson error structure.

^†^GLM or ^‡^GLMMs; error structures—b: binomial, qb: quasi-binomial, p: Poisson.

## Discussion

### Fungal communities associating with tree microsites and orchid roots

Knowing the presence as well as identity of OMF on microsites and within orchid roots can facilitate the process of selecting both suitable microsite and compatible orchid species for translocation attempts. By employing Illumina^®^ ITS-targeted sequencing approach on bark samples, we found several Ceratobasidiaceae- and Serendipitaceae-related taxa that are commonly associated with both terrestrial and epiphytic orchids^13,37^. Similarly, we detected both fungal taxa in the roots of native epiphytic orchids, as well as Tulasnellaceae-associated OMF. However, the latter was not detected on bark samples; this shortfall was likely due to the downstream effects of inhibitory compounds such as polysaccharides and phenolics, which are typically found in recalcitrant materials such as tree bark^56^. These inhibitors are known to negatively affect primer efficacy by disrupting the annealing process of the primer to the DNA template during amplification and/or sequencing process, i.e. competitive binding^57^. In this case, it is likely that the ITS1/ITS4-TUL primer combination is highly susceptible to this inhibitory effect. Nevertheless, these findings suggest that OMF that are associated with the study species may be present on urban roadside trees as well.

Non-rhizoctonia OMF were also discovered on microsites; although largely unknown, this fungal group may potentially form mycorrhizal associations with native epiphytic orchid species. For instance, the basidiomycetous fungal genera—*Mycena* (Agaricales; sequence identity = 99%) and *Marasmius* (Agaricales; sequence identity = 90%)—were previously reported to form mycorrhizal relationships with epiphytic orchids, including species from the genus *Dendrobium*^25,58–60^. A specific ascomycete genus, *Fusarium* (sequence identity = 99%), was also detected on bark samples. Although ascomycetes are rarely involved in orchid mycorrhizas^13^, Salifah *et al*.^61^ demonstrated that *Fusarium* sp. can establish functional mycorrhizae with *Grammatophyllum speciosum* Blume seeds. In this study, we chose a conservative approach of considering only rhizoctonia fungi (i.e. Ceratobasidiaceae, Serendipitaceae, and Tulasnellaceae) for analyses and excluded other possible OMF. Nevertheless, we hope to see more studies implicate OMF beyond the three rhizoctonia taxa, especially since increasingly more OMF, both basidiomycetous and ascomycetous fungi, are being discovered^37,62^. We also encourage future studies to include other primer sets that may improve the detection as well as coverage of OMF on bark samples.

Some orchid species may exhibit differing mycorrhizal specificity. We found five orchid species associated with only one OMF-OTU, suggesting high mycorrhizal specificity^25,63^. Conversely, three orchid species associated with multiple OMF-OTUs, of which two formed associations with more than one rhizoctonia taxa, suggesting low mycorrhizal specificity. We also discovered a pair of orchid species from the genus *Cymbidium* associating with the same *Serendipita* OMF-OTU. Possible similarities in orchid mycorrhizal fungal preference among closely related epiphytic species were also reported in Martos *et al*.^25^. Overall, knowledge on mycorrhizal specificity can influence a species’ suitability for *ex situ* conservation. It is likely that species with low mycorrhizal specificity are preferable due to their less restrictive mycorrhizal preference^25^. Our evaluation of orchid-mycorrhizal specificity was based on roots sampled from translocated orchids only and thus may be limited. As the study species were part of Singapore’s “orchid conservation programme”, root sampling was constrained as well. Therefore, to improve the assessment of orchid-mycorrhizal specificity as well as the detection and coverage of OMF, we encourage prospective studies to involve more root samples collected from multiple sites, ideally (if possible), both natural and non-natural areas.

We also discovered non-mycorrhizal taxa that may benefit orchid development. Members of saprotrophic Hypocreales, Helotiales, and Pleosporales were identified on bark and root samples. These non-mycorrhizal taxa, which are frequently found within orchid roots, have been reported to indirectly amplify nutrient access to orchids via decomposition of local substrates^64–66^. However, the exact functions and effects of non-OMF on orchid physiology remain largely unknown and warrant further research attention^13,67^.

### Mycorrhizal distribution and niche requirements

OMF were widespread, occurring at majority of the study sites. We found that surrounding land use influenced OMF presence, with grassland habitat-types having the highest frequency of OMF occurrence and richness. This occupancy pattern may imply that dispersal and subsequent establishment of orchid mycorrhizal fungal spores/inocula are largely unhindered at sites that are surrounded by large, open areas—in this case, managed turf—unlike forests, in which wind velocities are typically reduced^68,69^.

In general, OMF presence and richness were correlated with presence of humus, a substrate derived from decomposed organic matter such as dead plant parts, stemflow leachates, and plant exudates^70,71^. Humus commonly accumulates at tree forks, forming vital sources of nutrients and water for mycelial survival and growth^23,72^. This may be the primary reason why the fork microsite, which is structurally ideal for aggregation of organic debris, had the highest frequency of OMF occurrence. On the other hand, we observed OMF richness to be highest at the branch microsite. Given that tree branches tend to be exposed and structurally angled (cf. fork and vertical stem), it is possible that this part of the tree has the highest capacity for interception of diverse fungal spores/inocula and rain^73^. The open structure and orientation of tree branches however may not be ideal for humus accumulation (cf. fork); this may perhaps be the reason why the frequency of OMF occurrence on branch microsites was slightly lower than fork microsites.

Our results denote the central role of precipitation levels in determining OMF presence and richness at both microsite and site scale. Fungal establishment and mycelial development are particularly dependent on moisture conditions^72^; Querejeta *et al*.^74^ noted that fungal mortality increases with lower moisture, particularly during dry periods. Presence of mosses can enhance the water-holding capacity of microsites^75,76^. The association between richness of Serendipitaceae-associated OMF and both rainfall and moss presence suggests that members of this fungal taxon may be more sensitive to moisture limitation than that of Ceratobasidiaceae-associated OMF. The additional dependence on moisture conditions may explain their lower frequency of occurrence and richness, as well as limited distribution, as compared to Ceratobasidiaceae-associated OMF.

Overall, our findings indicate that OMF presence and richness vary in accordance to precipitation levels and humus presence. Both biophysical factors are also known to influence orchid germination and development^25,26,77^, suggesting possible overlap between niche requirements of OMF and orchid species. Such OMF-orchid correspondence can benefit the microsite selection process, therefore improving the likelihood of a successful translocation.

### Potential applications in orchid conservation

Our findings demonstrate the widespread presence and distribution of OMF-associated (micro)sites as well as their potential as orchid habitats. Based on the orchid-site suitability assessment, the similarities in orchid mycorrhizal fungal associations between bark and orchid root samples may suggest high amenability of native orchids towards translocation efforts, especially for species with low mycorrhizal specificity^20,78^. Such species are able to associate with different fungal lineages, and possibly multiple OMF partners, which can exploit various nutritional resources, and as a consequence, enhance the nutrient uptake of host orchid^66,79,80^. Orchid species that showed high mycorrhizal specificity may be advantageous for orchid conservation as well. Unique orchid-fungal affiliations may induce spatial differentiation among establishment sites (at tree or site scale), leading to possible co-occupancy of multiple species^81,82^.

The geographic distribution of OMF at landscape scale highlights the prevalence of sites suited for orchid species that associated with Ceratobasidiaceae-related OMF; thus, it might be sensible to focus on these species in future translocation attempts. Although more limited, Serendipitaceae-associated sites appear to have greater potential for orchid conservation as this fungal taxon was linked with five study species. To maximise this potential, we propose fungal inoculations on microsites that meet the niche requirements of Serendipitaceae-associated OMF, i.e. presence of humus, moss, and high precipitation levels (see McCormick *et al*.^47^). In fact, this may be a good pre-translocation practice for any applicable species. Since high-throughput sequencing-based quantification of species abundance is prone to overestimation^83^, we did not quantify the orchid mycorrhizal fungal density/potential (abundance and vitality) of each site. Nevertheless, fungal density/potential can influence a microsite’s capacity to support orchid establishment and development and therefore should be considered in future research^24,47^.

This study demonstrates that translocated orchids can form relationships with compatible OMF on host trees in non-forest habitats, in this case, urban parks (root sampling sites). De hert *et al*.^84^, Keel *et al*.^85^, and Waud *et al*.^78^ observed mycorrhizal associations in germinated seeds that were sowed in novel, unoccupied habitats, i.e. sites without adults. Similarly, Downing *et al*.^20^ detected OMF in orchids that were translocated beyond their natural ranges. These evidence suggest that communities of OMF in non-natural habitats, including urban landscapes, can support orchid establishment, survival, as well as development and thus should be considered in future *ex situ* conservation efforts. Ideally, these fungal communities should have the capacity to support both germinating seeds and mature orchids^86^.

This is the first study to explore OMF presence and distribution, as well as assess the biophysical factors that influence both aspects, on epiphytic microsites directly via bark samples, rather than indirectly via orchid roots. By employing metabarcoding and high-throughput sequencing, we were able to screen a large number of environmental samples and characterise fungal communities at a much higher resolution than before, thus making it an efficient and valuable management tool for large-scale detection of OMF, i.e. potential orchid colonisation sites. This tool may be particularly useful in dynamic or disturbed habitats whereby OMF are stochastically available^87–89^. Absence of obligate OMF must be dealt with if orchid conservation programmes are to be successful with lasting conservation benefits^90^. Applicable countermeasures include fungal isolation, preservation, and propagation as well as on-site inoculation of relevant fungal cultures^20,47,91,92^. Cevallos *et al*.^93^ noted the influence of keystone species: a core OMF species in which mycorrhizal communities are built around. Given their fundamental role in mycorrhizal assembly, future conservation strategies should perhaps prioritise keystone OMF and regularly monitor (e.g., via molecular detection) for their continued presence^13^.

Ultimately, knowledge on specific OMF—primarily presence, geographical distribution, and niche requirements—is imperative for future plans of establishing orchids, especially endangered species, in either natural or urban landscapes^13,24,94^. Knowledge on another biotic dependency of orchids, specific pollinator(s), is equally crucial for orchid propagation and therefore continuity^15^. Hence we encourage future studies to focus on orchid-pollinator interaction as well, especially in urban habitats. With appropriate planning, urban environments can serve as viable habitats for epiphytic orchid species, and accordingly, help mitigate their extinction risks.

## Methods

### Species selection

We focussed on 11 native species (planted as part of the “orchid conservation programme”; see Yam *et al*.^23^ for descriptions); except for *Bulbophyllum vaginatum* (Lindl.) Rchb. F. (nationally Endangered), *C. bicolor* (nationally Critically Endangered), and *C. finlaysonianum* (nationally Critically Endangered), all other species—*Bulbophyllum blumei* (Lindl.) J. J. Sm., *B. medusae*, *Coelogyne mayeriana* Rchb. f., *C. rochussenii*, *D. aloifolium*, *D. leonis*, *G. speciosum*, and *P. cornu-cervi*—are Presumed Nationally Extinct^95^ (see Supplementary Table S1). The average number of years since planting of all orchid species is 4.2 years. These species were selected primarily for three reasons: (1) differing conservation statuses, (2) easy access by bucket crane/ladder for root sampling, and (3) consistency in host tree, as all individuals were planted on *Albizia saman* trees. *Albizia saman*—native to South America—is the most widely planted urban tree in Singapore^96^. It has distinctive characteristics such as wide, umbrella-shaped crown, leaf-free inner branches, and flaky bark that make this species favourable host trees for epiphyte colonisation^19^.

### Sample collection

Through collaboration with Singapore’s National Parks Board, we generated a comprehensive list of roads planted with *Albizia saman* trees. From this list, 30 roads/sites were randomly selected, ten in each habitat stratum (“habitat-type”) defined by the dominant surrounding land use type^17^ (Fig. 1): urban (surrounded by man-made structures), grassland (surrounded by large areas of manicured turf grass), or secondary forest (surrounded by tree-dominated urban parks, lowland tropical forest, or young secondary forest patches occurring on previously degraded or cleared land)^97^. The dominant land use was defined as >50% of the area within an octagon of ~ 200 m diameter, drawn in Google Earth around the selected site (see Supplementary Fig. S1). At each site, three individual trees were randomly selected and the location of each tree was recorded using a Garmin^®^ GPSMAP 60CSx GPS receiver (Garmin International, Inc., Olathe, KS, USA).

To quantify the presence of OMF on roadside trees, we sampled bark (~25 cm^2^ per sample) from 90 urban trees in September 2016. For each tree, sample collection points within each of the three microsites—stem, fork, and branch—were randomly selected (total microsites = 270). We then employed a grid system and random number generator to select a specific quadrat for bark collection. Each individual sample was placed in a storage bag and stored at -80 °C prior to DNA extraction.

We sampled roots of translocated orchids to identify species-specific OMF (independent of bark samples). Approximately 5–6 orchid roots were collected from six different individuals per species. To avoid cross-contamination, root samples were collected using a sterilised scalpel and rubber gloves, placed in separate storage bags, and stored at -80 °C until DNA extraction. In total, 65 samples were collected from four different urban parks (see Supplementary Table S1); one *B. medusae* individual was not collected during the sampling period due to tree pruning.

Several biophysical variables were recorded at each tree: microsite location (stem/fork/branch), substrate (presence/absence of humus and moss), tree DBH (diameter at breast height; measured with a diameter tape, at 1.3 m above the base), mean ambient temperature, mean rainfall, and mean wind speed. Climate data were 5-year daily averages, calculated from collated weather records provided by Meteorological Service Singapore^98^. We also measured the nearest distance between individual trees located on the same road and distance of trees from nearest forest vegetation via Google Earth. Bark chemistry (e.g., pH, hydrocarbon content) was not quantified and analysed in this study.

### Molecular analyses

To extract fungal DNA from tree bark, the material of each sample was removed by scraping the surface with sterile scalpel. Approximately 50 mg of this material was ground using liquid nitrogen, sterile mortar, and pestle, followed by bead-based homogenisation in Bead Ruptor 24 (Omni International, Kennesaw, GA, USA). To extract fungal DNA from orchid roots, the root material was cut into 5 cm pieces, washed with Milli-Q^®^ water (Merck & Co., Inc., Kenilworth, NJ, United States) for 2 min, surface-sterilised in 1% sodium hypochlorite for 1 min, and rinsed with Milli-Q^®^ for 2 min. Six to eight root sections per plant were pooled (~50 mg) and homogenised using Bead Ruptor 24.

Total DNA was extracted from both homogenised bark and root samples using a modified CTAB protocol^99^. The fungal nuclear ribosomal internal transcribed spacer (ITS) sequences were amplified using primer combinations ITS86F/ITS4^100^ and ITS1/ITS4-TUL^101^. Each primer was tagged with a 9 bp long sequence for specimen-to-sequence association and a dual indexing strategy was used. Two replicates of polymerase chain reaction (PCR) amplifications were conducted for each DNA extract. PCRs were conducted in 25 µl reaction volumes containing 10 µl of 100-times diluted DNA extract and 15 µl of GoTaq^®^ Colorless Master Mix (Promega Corporation, Madison, WI, USA). PCR temperature profile for ITS86F/ITS4 primer-pair was as follows: initial denaturation step at 94 °C for 3 min, followed by 30 cycles of denaturation at 94 °C for 45 s each, annealing at 59 °C for 45 s, and extension at 72 °C for 2 min. For ITS1/ITS4-TUL primer-pair, PCR conditions were as follows: initial denaturation step at 96 °C for 2 min, followed by 35 cycles of denaturation at 94 °C for 30 s each, annealing at 60 °C for 40 s, and extension at 72 °C for 1 min. The final cycle of both amplifications was followed by a 7-min extension at 72 °C. PCR products were verified on 1% agarose gel, quantified using Qubit^®^ 2.0 Fluorometer (Thermo Fisher Scientific, Waltham, MS, USA), pooled in equimolar quantities, and cleaned using MinElute PCR Purification Kit (Qiagen, Hilden, Germany). The pooled products were subjected to paired-end (2 × 300 bp) sequencing on an Illumina^®^ MiSeq sequencer (Illumina, Inc., San Diego, California, USA). Libraries were prepared using NEB Ultra II DNA Library Preparation and amplification-free protocol (New England BioLabs, Massachusetts, USA).

The sequence reads were merged using PEAR version 0.9.11^102^ and processed using OBITools version 1.2.11^103^. Samples and replicates were demultiplexed using the *ngsfilter* command; only amplified regions remained for subsequent analyses. We then employed the *obiuniq* command to identify and cluster strictly identical sequences. Sequences with <1% sample-specific percentage occurrence were removed (potential amplification/sequencing errors). Sequences shorter than 100 bp were also omitted via the *obigrep* command. Given that there were two replicates of PCRs per sample, only sequences that passed the various filtering criteria in both replicates were retained for further analyses. The sequences were then aligned and clustered into operational taxonomic units (OTUs) at 97% sequence similarity criterion using VSEARCH version 2.6.0^104^. To identify the different OTUs, BLAST searches were performed on representative sequences to determine the closest match represented in GenBank (*nt* database). No ties—i.e. same OTU sequence giving hit to multiple species at same best identity—were encountered. A taxonomic assignment was given for a match of ≥85% identity (as done in Xing *et al*.^105,106^). Based on current orchid mycorrhizal fungal knowledge, only OTUs related to rhizoctonia taxa (i.e. Ceratobasidiaceae, Sebacinales/Serendipitaceae, Tulasnellaceae; see Dearnaley *et al*.^13^ and Jacquemyn *et al*.^27^) were considered potentially mycorrhizal (i.e. OMF-OTU). Other OTUs were considered as non-rhizoctonia OTUs, which are endophytic fungi that associate with orchid roots that may or may not provide benefit to the host^107^. Note that we also tested sequence identification using *ecotag* in OBITools and EMBL database as well as BLAST to UNITE database, but overall, GenBank yielded better matches.

Additionally, orchid-site suitability was assessed based on similarity of fungal taxa at genus-level, i.e. generic correspondence between bark- and root-associated OMF-OTUs. Thus, if a specific OMF genus was detected on a bark sample—a representative of site—as well as in an orchid root sample, we considered the site to be a potential habitat for the orchid species. We chose to compare fungal genera rather than species because (1) based on a recent large-scale study by Martos *et al*.^25^, tropical epiphytic orchids tend to have low mycorrhizal specificity, mainly associating with typical genera/species found in the three rhizoctonia families and (2) the fungal databases consist of numerous poor-quality fungal sequences—particularly species-level sequences—with low-resolution taxonomic annotations and subpar technical quality^108,109^. Hence to achieve a reasonable yet conservative compromise, a genus-based comparative approach was adopted.

### Statistical analyses

A non-metric multidimensional scaling (NMDS) analysis based on the Bray-Curtis dissimilarity matrix was used to visualize variation in orchid mycorrhizal fungi between habitat-types^110^; we then performed a variance analysis of these distances using analysis of similarities (ANOSIM)^111^. We also used the nonparametric diversity estimator Chao2^112^ to estimate the OMF richness of the microsite-level data set.

We applied either binomially/Poisson-distributed generalised linear models (GLMs) or generalised linear mixed models (GLMMs)^113^ to explore the correspondence of biophysical factors with (1) the presence of OMF (any OMF-OTU) at the microsite level, (2) OMF richness (i.e. number of OMF-OTUs) at the microsite-level, and (3) frequency of OMF occurrence (i.e. total number of microsites with OMF-OTUs per site) at the site-level. Multicollinearity was assessed and highly collinear variables were removed from the models. All models were conducted at two levels: (1) overall (i.e. all orchid mycorrhizal fungal families) and (2) family-specific. When overdispersion was evident, the model was fitted with quasi-binomial or quasi-Poisson error structure. The explanatory variables for the models were all recorded biophysical variables. “Tree” was a random factor for all GLMMs. Akaike’s information criterion (AIC)^114^ was employed for step-wise simplification and model evaluation. Additionally, GLM deviance was estimated as goodness-of-fit. Spatial autocorrelation was examined using correlograms and variograms^115^.

NMDS, ANOSIM, and diversity estimator test were conducted on PAST Version 3.17^116^. All other analyses were conducted using R version 3.4.2^117^, including the packages “spatial”^118^ and “car”^119^.

## Supplementary information

Izuddin et al., 2019 - Supplementary information

## Data Availability

The datasets generated during the current study are available in the figshare repository, 10.6084/m9.figshare.8063366.v1.
