# Supplementary material for: Availability of orchid mycorrhizal fungi on roadside trees in a tropical urban landscape
Source: Sci Rep. 2019 Dec 20;9:19528. doi: 10.1038/s41598-019-56049-y (PMC6925147; doi:10.1038/s41598-019-56049-y)
Supplement: Supplementary file 1 — Izuddin et al., 2019 - Supplementary information [file 41598_2019_56049_MOESM1_ESM.pdf]

Supplementary information

Supporting information to the paper Izuddin, M.[1,3], Srivathsan, A.[1], Lee, A. L.[1], Yam, T. W.[2], & Webb, E. L.[1,3], Availability of orchid mycorrhizal fungi on roadside trees in a tropical urban landscape, Scientific Reports.

[1]Department of Biological Sciences, National University of Singapore, 14 Science Drive 4, Singapore 117543

[2]Singapore Botanic Gardens, 1 Cluny Road, Singapore 25956

[3]Corresponding authors: izuddin@u.nus.edu, ted.webb@nus.edu.sg

Supplementary Fig. S1 Examples of site classification based on dominant surrounding land use, i.e. "habitat-type", from left: secondary forest (Orchard Boulevard), grassland (Boon Keng Road), and urban (Gul Road). Diameter of octagons is 200 m (drawn to scale). All satellite images were obtained from Maxar Technologies, Google™ Earth version 7.1 (https://www.google.com/earth/).

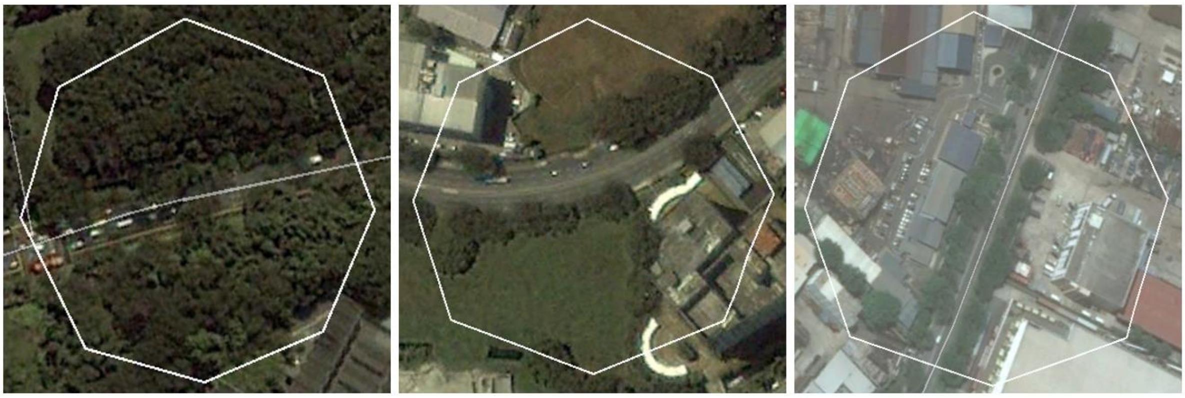

Supplementary Table S1 List of surveyed and sampled epiphytic native orchids in this study. $Planted_{yrs}$ = average number of years since planting. Nomenclature and conservation statuses are based on Chong et al.[95].

Species	Conservation status	$Planted_{yrs}$	Detection of OMF
Bulbophyllum blumei (Lindl.) J. J. Sm.[B,C,G,R]	Extinct	4.3	No
Bulbophyllum medusae (Lindl.) Rchb. f.[C,G,R]	Extinct	4.3	Yes
Bulbophyllum vaginatum (Lindl.) Rchb. f.[B,C,G,R]	Endangered	4.5	No
Coelogyne mayeriana Rchb. f.[B,C,G,R]	Extinct	4.3	Yes
Coelogyne rochussenii de Vr.[C,R]	Extinct	3.8	Yes
Cymbidium bicolor ssp. Lindl. ssp. *pubescens* (Lindl.) Du Pay & Cribb[B,G]	Critically Endangered	4.6	Yes
Cymbidium finlaysonianum Lindl.[B,C,G,R]	Critically Endangered	4.5	Yes
Dendrobium aloifolium (Blume) Rchb. f.[R]	Extinct	3.8	Yes
Dendrobium leonis (Lindl.) Rchb. f.[B,C,G,R]	Extinct	4.3	Yes
Grammatophyllum speciosum Blume[B,C,R]	Extinct	4.5	No
Phalaenopsis cornu-cervi (Breda) Blume & Rchb. f.[C,R]	Extinct	3.8	Yes

Sampling sites (urban parks): [B]Bukit Batok Nature Park (n = 12), [C]Changi Beach Park (n = 16), [G]Pasir Ris Park (Pasir Ris Green = 12), [R]Pasir Ris Park (Pasir Ris Road = 25)
